# An Unexpected Inverse Relationship Between Biofilm Formation and Antibiotic Resistance in *Stenotrophomonas maltophilia*

**DOI:** 10.3390/antibiotics15010085

**Published:** 2026-01-15

**Authors:** Arianna Pompilio, Giovanni Di Bonaventura

**Affiliations:** 1 Department of Medical, Oral, and Biotechnological Sciences, “G. d’Annunzio” University of Chieti-Pescara, 66100 Chieti, Italy; arianna.pompilio@unich.it; 2 Center of Advanced Studies and Technology, “G. d’Annunzio” University of Chieti-Pescara, 66100 Chieti, Italy

**Keywords:** *Stenotrophomonas maltophilia*, biofilm formation, antibiotic resistance, cystic fibrosis, multidrug resistance, bacterial persistence

## Abstract

**Background/Objectives**: *Stenotrophomonas maltophilia* is an opportunistic pathogen causing severe infections, particularly in patients with cystic fibrosis (CF). Its intrinsic multidrug resistance and biofilm-forming capacity complicate treatment. Although biofilms are generally associated with antimicrobial tolerance, the relationship between biofilm formation and planktonic antibiotic resistance in *S. maltophilia* remains poorly understood. This study investigated the association between antibiotic resistance profiles and biofilm production in clinical isolates from CF and non-CF patients. **Methods**: A total of 86 clinical isolates (40 from CF airways and 46 from non-CF patients) were analyzed. Susceptibility to seven antibiotics was assessed by disk diffusion, and multidrug resistance profiles were defined using standard criteria. Biofilm formation was quantified after 24 h using a crystal violet microtiter plate assay and categorized by using a semiquantitative scale. **Results**: High resistance rates were observed, particularly to meropenem (87.2%), ciprofloxacin (80.2%), and rifampicin (72.1%). CF isolates exhibited significantly higher resistance to piperacillin/tazobactam and a greater prevalence of multidrug resistance. Biofilm formation was detected in 94.2% of isolates, with strong or powerful producers predominating. However, CF isolates formed significantly less biofilm than non-CF isolates. Notably, resistance to piperacillin/tazobactam and meropenem was associated with reduced biofilm biomass and a lower proportion of high biofilm producers. Across all isolates, an inverse correlation was observed between the number of antibiotic resistances and biofilm biomass. These trends persisted after stratification by clinical origin, although some comparisons did not reach statistical significance. **Conclusions**: This study reveals an unexpected inverse relationship between planktonic antibiotic resistance and biofilm-forming capacity in *S. maltophilia*. Enhanced biofilm production may represent an alternative persistence strategy in more antibiotic-susceptible strains, with important implications for infection management and therapeutic failure.

## 1. Introduction

Once regarded as a low-virulence microorganism, *Stenotrophomonas maltophilia* has emerged as a clinically relevant opportunistic pathogen responsible for a broad range of infections involving multiple organ systems, including the respiratory, gastrointestinal, and urinary tracts. Clinical manifestations include pneumonia, catheter-associated bacteremia and septicemia, osteochondritis, mastoiditis, meningitis, and endocarditis [1]. The bacterium is particularly prevalent in patients with cystic fibrosis (CF), in whom it is frequently isolated from the respiratory tract, with reported prevalence rates ranging from approximately 10% to 30% [2].

The treatment of *S. maltophilia* infections remains a major clinical challenge because of the bacterium’s extensive intrinsic and acquired antibiotic resistance mechanisms, including the chromosomally encoded L1 and L2 β-lactamases and multiple multidrug efflux pump systems (e.g., SmeDEF), which together confer resistance to a wide range of broad-spectrum antimicrobial agents [3]. In addition, *S. maltophilia* readily forms biofilms on both abiotic and host tissues, a phenotype that further compromises the efficacy of clinically relevant antibiotics, including aminoglycosides, fluoroquinolones, and tetracyclines [4,5,6].

Biofilm growth is widely recognized as a major contributor to antimicrobial tolerance because it limits antibiotic penetration, promotes antibiotic inactivation, and fosters physiological heterogeneity within bacterial populations [7,8]. The elevated cell density and oxidative stress characteristics of biofilms can increase mutation rates and facilitate horizontal gene transfer [9]. Compared with their planktonic counterparts, bacteria in biofilms exhibit greater resistance to nutrient starvation, pH fluctuations, and oxidative stress [10]. Biofilms may also increase resistance by altering the expression of pre-existing antibiotic resistance genes [11] and by increasing the proportion of tolerant or persister cells within the population, due to reduced bacterial metabolic activity within the biofilm interior [12].

Despite extensive evidence linking biofilm formation to increased antibiotic tolerance, the relationship between biofilm-forming capacity and antibiotic resistance in *S. maltophilia* planktonic cells remains poorly defined, leaving unresolved whether strong biofilm formation is consistently associated with increased planktonic resistance or whether trade-offs between these phenotypes may exist. Moreover, potential differences between isolates from CF and non-CF clinical settings have not been systematically explored.

In this study, we address these gaps by conducting a comparative analysis of a large and diverse collection of *S. maltophilia* clinical isolates obtained from the airways of CF patients and from multiple anatomical sites in non-CF patients. By jointly assessing biofilm-forming ability and efficiency, planktonic antibiotic resistance profiles, and clinical origin, our work provides new insight into the interplay among these traits, revealing an unexpected relationship between biofilm formation and antibiotic resistance and setting the stage for the results presented below.

## 2. Results

### 2.1. Antibiotic Resistance

Antibiotic susceptibility testing results for the 86 isolates tested (40 from CF patients and 46 from non-CF patients) are summarized in Table 1 and Table S1 (Supplementary Materials). Overall, resistance to meropenem, ciprofloxacin, rifampicin, piperacillin/tazobactam, chloramphenicol, levofloxacin, and cotrimoxazole was observed in 87.2%, 80.2%, 72.1%, 50%, 47.7%, 26.7%, and 18.6% of isolates, respectively (Table 1). Stratified by patient type, CF isolates exhibited a significantly higher resistance rate to piperacillin/tazobactam than non-CF isolates (90.0 vs. 52.2%; *p* = 0.0001) (Table 1).

Regarding resistance to multiple antimicrobial agents, the multidrug-resistant (MDR) phenotype was observed in a significantly higher proportion of CF isolates than of non-CF isolates (97.5 vs. 67.4%, respectively; *p* = 0.0002) (Table 1). CF isolates also exhibited higher rates of the extensively drug-resistant (XDR) and pan-drug-resistant (PDR) phenotypes than the non-CF group, although the differences were not statistically significant (XDR: 60% vs. 54.3%; PDR: 15% vs. 4.3%, respectively, for CF and non-CF isolates) (Table 1).

The antibiotic resistance patterns showed that most isolates had a high frequency of MDR; specifically, 62 of 86 isolates (72.1%) were resistant to at least 6 of the 7 antibiotics tested. However, no differences in multi-resistance levels were observed between the CF and non-CF groups (Table 1).

### 2.2. Biofilm Formation

Results of the biofilm formation assay are summarized in Table S1 (Supplementary Materials). The cut-off for biofilm formation—i.e., ODc = mean OD of negative control + (3 × SD of negative control)—was 0.076. This indicated a weak biofilm producer if 0.076 < OD_492_ ≤ 0.152, a moderate producer if 0.152 < OD_492_ ≤ 0.304, a strong producer if 0.304 < OD_492_ ≤ 0.608, and a powerful producer if OD_492_ > 0.608.

Most *S. maltophilia* isolates tested (81 out of 86, 94.2%) formed biofilm, with strong and powerful biofilm producer classes being the most prevalent (40.7% and 33.7%, respectively; *p* values at least 0.0009 vs. other classes). However, trends varied by patient source.

Although CF and non-CF isolates showed comparable biofilm-forming capabilities (90% vs. 97.8%, respectively), CF isolates were less efficient (OD_492_, median: 0.395 vs. 0.615 for CF and non-CF isolates, respectively; *p* = 0.006). Confirming these findings, a significantly higher proportion of powerful biofilm producers was observed among non-CF isolates than among CF isolates (50% vs. 15%, respectively; *p* = 0.0007). Conversely, moderate biofilm producers were found more frequently among CF than non-CF isolates (22.5% vs. 2.2%, respectively; *p* = 0.005).

### 2.3. Correlation Between Antibiotic Resistance and Biofilm Formation

**Figure 1:**
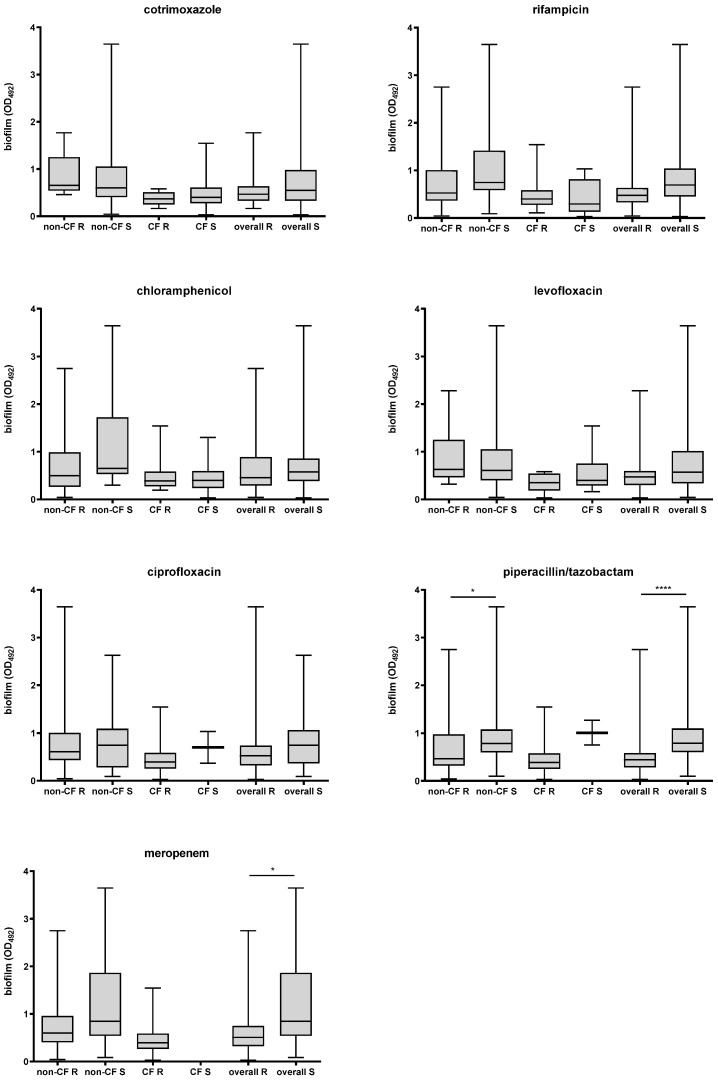
Biofilm formation of *S. maltophilia* according to susceptibility (S) or resistance (R) to several antibiotics, and stratified by patient type (CF, cystic fibrosis; non-FC, noncystic fibrosis). Results are shown as box-and-whisker plots; each box shows the median, with the bottom and top edges indicating the 25th and 75th percentiles, respectively, and the whiskers extend to the most extreme data points not considered outliers. Statistical significance by Mann–Whitney test: * *p* < 0.05, **** *p* < 0.0001.

Considering the isolates as a whole, those resistant to piperacillin/tazobactam or meropenem produced significantly less biofilm than susceptible isolates (median OD492; piperacillin/tazobactam: 0.446 vs. 0.793, *p* < 0.0001; meropenem: 0.598 vs. 0.847, *p* = 0.048, respectively, for resistant and susceptible isolates) (Figure 1). Confirming these findings, a significantly lower proportion of powerful producers was observed in piperacillin/tazobactam-resistant compared to susceptible isolates (18.3% vs. 69.2%, respectively; *p* = 0.0001). No significant differences were found for other antibiotics.

Stratified by patient type and isolation site, airway isolates from non-CF patients that were resistant to piperacillin/tazobactam produced less biofilm than susceptible isolates (OD_492_, median: 0.470 vs. 0.788, respectively; *p* = 0.012) (Figure 1). No significant differences were observed among the biofilm producer groups. Notably, the percentage of isolates categorized as strong biofilm producers was nearly double among susceptible isolates compared to resistant isolates (63.6% vs. 33.3%, respectively); however, this difference did not reach statistical significance due to the small sample size.

Among isolates collected from the airways of CF patients, the proportion unable to form biofilm was significantly lower among resistant than among susceptible isolates for meropenem (0% vs. 100%; *p* = 0.03), ciprofloxacin, and piperacillin/tazobactam (0% vs. 50%; *p* = 0.002).

**Figure 2:**
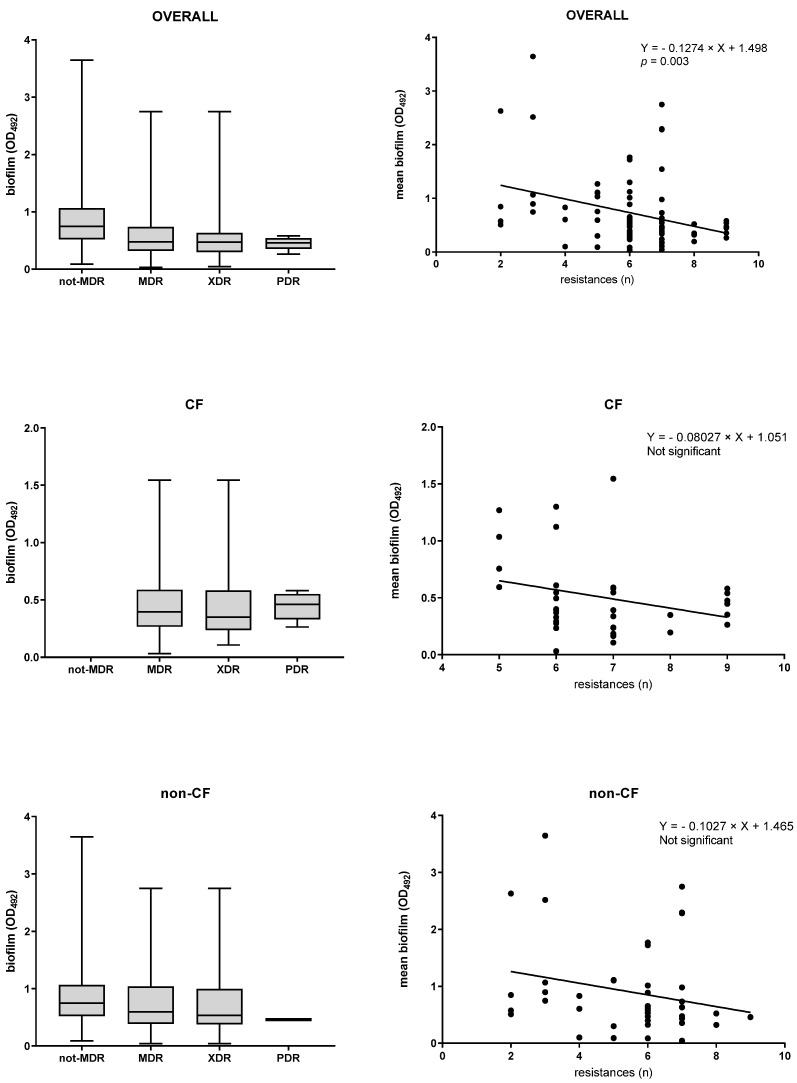
Biofilm formation and multidrug resistance (MDR, multidrug resistance; XDR, extensively drug resistance; PDR, pandrug resistance) in *S. maltophilia* isolated from cystic fibrosis (CF) and non-CF patients. **Left side**: results are shown as box-and-whisker plots (the central mark indicates the median, the bottom and top edges of the box indicate the 25th and 75th percentiles, respectively, and the whiskers extend to the most extreme data points not considered outliers). **Right side**: correlation between biofilm formation efficiency and antibiotic resistance level, as assessed by linear regression analysis.

No statistically significant differences in median biofilm amount were observed among non-MDR, MDR, XDR, and PDR isolates, regardless of the patient group considered (Figure 2). A similar trend was observed in the proportion of biofilm-producing groups among non-MDR, XDR, and PDR isolates. The percentage of non-MDR isolates classified as powerful producers was higher than that observed in MDR, XDR, and PDR isolates, although statistical significance was achieved only in the latter group (50% vs. 0%, *p* = 0.022; for non-MDR and PDR, respectively). The percentage of powerful and strong producers was comparable between MDR and XDR (MDR: 30% vs. 42.9%; XDR: 28.6% vs. 44.9%), but was significantly higher than in other groups (MDR: *p* at least 0.04 vs. other classes; XDR: *p* at least 0.004 vs. other classes). PDR isolates were not seen as powerful producers, while the proportion of strong producers was higher than that of moderate and non-producers (75% vs. 12.5% and 12.5%, respectively; *p* = 0.004).

The overall multidrug resistance level—i.e., the number of resistances displayed by an isolate—was negatively associated with the amount of biofilm formed, as indicated by linear regression analysis (*p* = 0.003) (Figure 2). A similar trend was observed after stratification by CF and non-CF isolates; however, it did not reach statistical significance.

## 3. Discussion

Antibiotic resistance in *S. maltophilia* is an increasing concern, particularly in the lungs of people with CF, where its prevalence is rising. This study reported high levels of resistance to meropenem (87.2%), ciprofloxacin (80.2%), rifampicin (72.1%), piperacillin/tazobactam (50%), and chloramphenicol (47.7%), confirming previous studies [13]. Notably, CF isolates showed higher resistance to piperacillin/tazobactam than non-CF isolates. This finding likely reflects the frequent use of piperacillin/tazobactam as an antipseudomonal agent in patients with CF experiencing pulmonary exacerbations [14].

The antibiotic resistance of *S. maltophilia* was also indicated by the overall prevalence of the MDR phenotype, which was 81.4%. Interestingly, MDR isolates were more common in CF than in non-CF isolates. As is well known for *Pseudomonas aeruginosa*, the development of MDR in *S. maltophilia* lung isolates from CF patients can be attributed to its ability to adapt to the CF airway microenvironment through various genotypic changes and to develop mutational resistance under high selective pressure [15,16].

Our findings indicated that trimethoprim/sulfamethoxazole and levofloxacin were the most effective drugs tested. However, the susceptibility rates we observed (81.4% and 73.7%, respectively, for trimethoprim/sulfamethoxazole and levofloxacin) were lower than those reported in previous studies from other countries [17,18]. Rhee et al. [19] reported even higher resistance rates, over 30%, for both antibiotics. These findings indicate increasing resistance to the last-resort drug for treating multidrug-resistant *S. maltophilia* infections, underscoring the importance of robust control policies to limit the dissemination of resistant *S. maltophilia* strains and the need for further research to develop new treatments.

Most bacteria in nature exist in aggregated communities known as biofilms. Cells within a biofilm exhibit significant physiological changes compared with their planktonic counterparts [20]. Biofilms are associated with numerous infections that can severely impact patients [21]. Indeed, infections involving biofilm formation are often chronic and highly resistant to antibiotic therapy [21]. Several studies have shown that biofilms are crucial to the persistence of *S. maltophilia* healthcare-associated infections, especially in patients with mechanical ventilation devices and CF patients [22,23]. Our findings confirmed that *S. maltophilia* has a significant propensity to form biofilms [22]. Over 94% of isolates produced biofilm. Notably, most isolates exhibited high biofilm-forming efficiency and were classified as strong or powerful producers. Decreased efficiency is a distinctive feature of isolates from CF patients, as indicated by a significantly lower proportion of powerful biofilm producers and a higher proportion of moderate biofilm producers compared with isolates from non-CF patients. These findings confirm that *S. maltophilia* adapts to a stressed environment, such as the CF lung [24].

The correlation between antibiotic resistance and the biofilm-forming ability of planktonic cells has been studied in Gram-positive and Gram-negative pathogens [25,26,27], raising questions about the mechanisms underlying the balance between these biological phenomena. Here, we evaluated, for the first time, the potential relationship between antibiotic resistance and the biofilm-forming capacity of *S. maltophilia*, leading to several conclusions.

First, isolates resistant to piperacillin/tazobactam or meropenem produced less biofilm than susceptible isolates, as indicated by differences in median biofilm quantity and the prevalence of the high-producing class. A similar trend appears to be specific to non-CF isolates. In contrast to our findings, Liaw et al. [28], evaluating the roles of integrons, efflux pumps, SpgM, melanin, and biofilm in MDR among 40 clinical isolates of *S. maltophilia*, observed that MDR isolates formed biofilm more readily than non-MDR isolates. Additionally, high biofilm formation was more prevalent among resistant than among susceptible isolates to piperacillin/tazobactam, whereas no difference was observed with meropenem. Differences in growth conditions (i.e., Luria–Bertani rather than TSB) and susceptibility breakpoints (i.e., established by CLSI rather than EUCAST) may explain the discrepancies from our findings.

Second, the percentage of non-CF isolates classified as high biofilm producers was nearly double among susceptible isolates compared with resistant isolates, and the proportion of CF isolates unable to form biofilm was significantly lower among resistant isolates than among susceptible isolates for meropenem, ciprofloxacin, and piperacillin/tazobactam.

Third, although the prevalence of high- and strong-producer classes was higher among MDR and XDR isolates than among non-MDR isolates, the number of resistances exhibited by an isolate was negatively correlated with the amount of biofilm produced.

Together, these results indicate that in *S. maltophilia*, there is a negative correlation between antibiotic resistance and biofilm-forming efficacy. Biofilms are known to confer greater antibiotic resistance and enhanced host immunity on microorganisms. From this perspective, high biofilm-forming efficiency may be considered an alternative strategy that antibiotic-susceptible strains adopt to evade antimicrobial treatments and persist longer within the host [25]. This adaptive strategy could be responsible for unexplained treatment failures and recurrences in susceptible isolates [29].

## 4. Materials and Methods

### 4.1. Bacterial Strains

Eighty-six *S. maltophilia* isolates were investigated: 40 from respiratory specimens collected from CF patients and 46 from non-CF patients at different sites (i.e., 29 from the respiratory tract, 11 from blood, and 6 from other sources) (Table S1, Supplementary materials).

### 4.2. Antibiotic Susceptibility Tests

The agar disk-diffusion technique was used to evaluate the antibiotic susceptibility of *S. maltophilia* isolates as described by EUCAST [30]. Antibiotic discs used for susceptibility testing were meropenem (10 µg), ciprofloxacin (5 µg), rifampicin (5 µg), piperacillin/tazobactam (30/6 µg), chloramphenicol (10 µg), levofloxacin (5 µg), and trimethoprim/sulfamethoxazole (1.25/23.75 µg) (Liofilchem srl, Roseto degli Abruzzi, Italy). *Escherichia coli* ATCC 25922 and *Pseudomonas aeruginosa* ATCC 27853 were used as control strains.

Interpretation of zone diameters was based on the current EUCAST breakpoint [30]. When no EUCAST breakpoints were available, CLSI breakpoints were used [31]. In accordance with Magiorakos et al. [32], a bacterial isolate was considered non-susceptible to an antimicrobial agent when it tested as resistant, intermediate, or non-susceptible using clinical breakpoints as interpretive criteria provided by the EUCAST or CLSI. Multidrug resistance (MDR) was defined as nonsusceptibility to ≥1 agent in ≥3 antimicrobial categories; extensively drug resistance (XDR) as susceptibility limited to ≤2 categories; pan drug resistance (PDR), as nonsusceptibility to all agents in all antimicrobial categories [32].

### 4.3. Biofilm Formation Assay

The ability of each isolate to form biofilm was assessed in a 96-well microtiter plate assay after 24 h of incubation at 37 °C and quantified as optical density at 492 nm (OD_492_) using a crystal violet colorimetric assay, as previously described [22]. The cut-off value for biofilm formation (ODc) was calculated as three standard deviations (SD) above the mean OD of the negative control: ODc = average OD of negative control + (3 × SD of negative control). Negative values were recorded as zero, and any positive value indicated biofilm production. Isolates were classified according to Stepanovic et al. [33] with minor modifications: OD ≤ ODc = no biofilm producer; ODc < OD ≤ 2 × ODc = weak biofilm producer; 2 × ODc < OD ≤ 4 × ODc = moderate biofilm producer; 4 × ODc < OD ≤ 8 × ODc = strong biofilm producer; and 8 × ODc < OD = powerful biofilm producer.

### 4.4. Statistical Analysis

Each experiment was performed in triplicate and repeated twice (n = 6). The D’Agostino & Pearson normality test indicated that the data were not normally distributed. Therefore, the Mann–Whitney test was used to evaluate differences in median biofilm biomass between the CF and non-CF groups and between susceptible and resistant isolates. Fisher’s exact test was used to evaluate differences in proportions. The correlation between biofilm formation efficiency and antibiotic resistance level was assessed using linear regression. Statistical analyses were performed using Prism software, version 7 (GraphPad Software, Boston, MA, USA), with *p*-values < 0.05 considered statistically significant.

## 5. Conclusions

This study demonstrates an unexpected inverse relationship between planktonic antibiotic resistance and biofilm-forming capacity in *S. maltophilia*. Clinical isolates with higher levels of resistance, particularly to piperacillin/tazobactam and meropenem, and with increasing multidrug resistance generally produced less biofilm, whereas more antibiotic-susceptible isolates exhibited enhanced biofilm formation. These findings suggest that biofilm formation may serve as an alternative persistence strategy in susceptible strains rather than merely accompany increased resistance.

Clinically, this underscores the limitations of relying solely on planktonic susceptibility testing to predict therapeutic outcomes in *S. maltophilia* infections, as strong biofilm producers may persist and cause treatment failure despite apparent susceptibility. Further studies are needed to elucidate the molecular basis of this trade-off and to assess its implications for infection management and the development of antibiofilm-based therapeutic strategies.

## Figures and Tables

**Table 1:** Frequency of antibiotic-resistance and multidrug-resistance phenotypes among *S. maltophilia* strains from patients with cystic fibrosis (CF) and patients without cystic fibrosis (non-CF).

	Overall (n = 86)		CF (n = 40)		non-CF (n = 46)		Fisher’s Exact Test (*p*; CF vs. non-CF)
**Antibiotic**	n	%	n	%	n	%	
meropenem	75	87.2	38	95.0	37	80.4	NS ^a^
ciprofloxacin	69	80.2	36	90.0	33	71.7	NS
rifampicin	62	72.1	33	82.5	29	63.0	NS
piperacillin/tazobactam	49	57.0	36	90.0	24	52.2	0.0001
chloramphenicol	41	47.7	18	45.0	24	52.2	NS
levofloxacin	23	26.7	13	32.5	10	21.7	NS
cotrimoxazole	16	18.6	10	25.0	6	13.0	NS
**Multidrug-resistant phenotypes**							
MDR	70	81.4	39	97.5	31	67.4	0.0002
XDR	49	56.9	24	60	25	54.3	NS
PDR	8	9.3	6	15	2	4.3	NS

^a^ NS, not significant.

## Data Availability

Data is unavailable due to privacy and ethical restrictions.

## Supplementary Materials

The following supporting information can be downloaded at: https://www.mdpi.com/article/10.3390/antibiotics15010085/s1. Table S1. Antibiotic susceptibility profiles, multidrug resistance classification, and biofilm-forming capacity of 86 *Stenotrophomonas maltophilia* clinical isolates (46 from non-cystic fibrosis patients and 40 from cystic fibrosis patients).

## Abbreviations

The following abbreviations are used in this manuscript:

CF	Cystic fibrosis
OD	Optical density
MDR	Multidrug resistance
XDR	Extensively drug resistance
PDR	Pandrug resistance
EUCAST	European committee for antimicrobial susceptibility testing
CLSI	Clinical laboratory standards institute
