# Supplementary material for: An Unexpected Inverse Relationship Between Biofilm Formation and Antibiotic Resistance in Stenotrophomonas maltophilia
Source: Antibiotics (Basel). 2026 Jan 15;15(1):85. doi: 10.3390/antibiotics15010085 (PMC12838282; doi:10.3390/antibiotics15010085)
Supplement: Supplementary file 1 [file antibiotics-15-00085-s001.zip › antibiotics-4089794-supplementary.pdf]

Table S1. Antibiotic susceptibility profiles, multidrug resistance classification, and biofilm-forming capacity of 86 *Stenotrophomonas maltophilia* clinical isolates (46 from non-cystic fibrosis patients and 40 from cystic fibrosis patients). Highlighted in red are intermediate or resistance results.

				Susceptibility to:										
**Hospital**	**Patient**	**Specimen**	**ID strain**	**SXT**	**RIF**	**CHL**	**LVX**	**CIP**	**TZP**	**MEP**	**MDR**	**XDR, PDR**	**mean biofilm (OD_{492})**	**biofilm class**
Pescara	non-CF	urine	SM 4	R	S	I	I	R	S	R	MDR	XDR	0.732	powerful
Pescara	non-CF	sputum	SM 5	R	R	I	I	I	I	R	MDR	PDR	0.000	non producer
Pescara	non-CF	sputum	SM 6	S	S	S	S	S	S	R	MDR		2.517	powerful
Pescara	non-CF	urine	SM 8	R	S	S	I	R	S	I	MDR	XDR	1.767	powerful
Pescara	non-CF	sputum	SM 14	S	S	S	S	S	S	S			0.847	powerful
Pescara	non-CF	sputum	SM 19	S	S	S	S	S	S	S			2.629	powerful
Pescara	non-CF	sputum	SM 29	S	R	S	S	I	R	R	MDR	XDR	1.720	powerful
Pescara	non-CF	sputum	SM 30	S	S	S	S	R	S	R			0.604	strong
Pescara	non-CF	sputum	SM 31	S	S	R	S	S	I	R			0.090	weak
Pescara	non-CF	sputum	SM 32	S	S	S	S	S	S	S			0.509	strong
Pescara	non-CF	sputum	SM 36	S	S	S	S	R	S	S			3.646	powerful
Pescara	non-CF	blood	SM 37	R	S	S	I	R	S	R	MDR		0.653	powerful
Pescara	non-CF	sputum	SM 38	S	S	S	S	S	S	I			0.896	powerful
Pescara	non-CF	sputum	SM 45	S	S	S	S	R	S	R			0.596	strong
Pescara	non-CF	blood	SM 46	S	I	I	S	I	S	R	MDR		0.888	powerful
Pescara	non-CF	sputum	SM 47	S	S	R	S	S	S	I			0.100	weak
Pescara	non-CF	blood	SM 48	S	I	S	S	R	S	I	MDR		0.299	moderate
Pescara	non-CF	vaginal swab	SM 49	S	S	R	S	S	S	S			0.746	powerful
Pescara	non-CF	sputum	SM 50	S	I	I	S	I	R	R	MDR	XDR	2.295	powerful
Pescara	non-CF	blood	SM 51	S	S	R	S	S	S	S			1.066	powerful
Rome	non-CF	sputum	SM 170	S	R	I	I	R	R	R		XDR	0.521	strong
Rome	non-CF	sputum	SM 171	S	R	S	S	I	I	S			1.099	powerful
Rome	non-CF	sputum	SM 172	S	R	R	S	R	R	R	MDR	XDR	0.432	strong
Rome	non-CF	sputum	SM 173	S	R	S	S	I	R	R	MDR	XDR	0.574	strong
Rome	non-CF	sputum	SM 174	S	R	S	S	I	R	R	MDR	XDR	0.532	strong
Rome	non-CF	sputum	SM 175	S	R	R	S	R	R	R	MDR	XDR	0.979	powerful
Rome	non-CF	sputum	SM 176	S	R	I	S	I	S	R	MDR	XDR	0.615	powerful
Rome	non-CF	sputum	SM 177	S	R	R	S	I	R	R	MDR	XDR	0.354	strong
Pescara	non-CF	sputum	SM 40	S	R	R	S	R	R	R	MDR	XDR	0.476	strong
Pescara	non-CF	vaginal swab	SM 39	R	R	I	I	R	R	R	MDR	PDR	0.459	strong
Pescara	non-CF	sputum	SM 42	S	I	S	S	I	I	R	MDR	XDR	0.323	strong
Pescara	non-CF	blood	SM 184	S	I	R	S	I	R	R	MDR	XDR	0.043	weak
Pescara	non-CF	rectal swab	SM 21	S	R	R	I	R	R	R	MDR	XDR	0.320	strong
Pescara	non-CF	blood	SM 183	S	I	S	S	I	R	R	MDR	XDR	0.397	strong
Pescara	non-CF	blood	SM 182	S	S	S	S	R	S	I			0.831	powerful
Pescara	non-CF	blood	SM 181	S	I	I	S	I	R	S	MDR	XDR	0.085	weak
Pescara	non-CF	blood	SM 186	S	S	S	S	S	S	S			0.575	strong
Pescara	non-CF	blood	SM 180	S	I	I	S	I	S	I	MDR	XDR	1.014	powerful
Pescara	non-CF	blood	SM 185	S	I	S	I	R	I	R	MDR	XDR	2.282	powerful
Pescara	non-CF	sputum	SM 10	S	R	R	S	I	R	R	MDR	XDR	0.633	powerful
Pescara	non-CF	sputum	SM 43	S	I	S	I	S	R	R	MDR	XDR	0.470	strong
Pescara	non-CF	sputum	SM 27	S	R	R	S	R	R	R	MDR	XDR	0.455	strong
Pescara	non-CF	sputum	SM 24	R	R	S	R	R	S	R	MDR	XDR	0.629	powerful
Pescara	non-CF	pharyngeal swab	SM 7	S	R	I	S	S	S	R	MDR		1.111	powerful
Pescara	non-CF	sputum	SM 143	S	R	R	S	R	I	R	MDR	XDR	2.750	powerful
Pescara	non-CF	sputum	SM 114	S	R	R	S	S	R	R	MDR	XDR	0.089	weak
Rome	CF	sputum	SM 103	S	R	S	S	R	R	R	MDR	XDR	0.400	strong
Rome	CF	sputum	SM 104	S	R	R	I	R	R	R	MDR	XDR	0.349	strong
Rome	CF	sputum	SM 105	R	R	R	I	R	R	R	MDR	PDR	0.581	strong
Rome	CF	sputum	SM 106	S	R	S	S	R	R	R	MDR	XDR	0.495	strong
Rome	CF	sputum	SM 107	S	I	S	S	R	R	R	MDR		0.593	strong
Rome	CF	sputum	SM 108	R	R	S	S	I	R	R	MDR	XDR	0.165	moderate
Rome	CF	sputum	SM 109	S	I	I	S	S	R	R	MDR		1.035	powerful
Rome	CF	sputum	SM 110	R	R	R	R	R	R	R	MDR	PDR	0.475	strong
Rome	CF	sputum	SM 111	S	S	S	I	R	R	R	MDR		0.031	weak
Rome	CF	sputum	SM 112	S	R	S	I	R	R	R	MDR	XDR	0.106	weak
Rome	CF	sputum	SM 113	R	S	S	S	R	S	S	MDR		0.000	non producer
Rome	CF	sputum	SM 115	S	R	S	S	I	R	R	MDR	XDR	0.329	strong
Rome	CF	sputum	SM 116	S	S	R	S	I	R	R	MDR	XDR	0.293	moderate
Rome	CF	sputum	SM 117	S	I	R	S	I	R	R	MDR	XDR	0.000	non producer
Rome	CF	sputum	SM 118	S	R	S	S	I	S	R	MDR		0.755	powerful
Rome	CF	sputum	SM 119	S	R	R	S	I	R	R	MDR	XDR	0.236	moderate
Rome	CF	sputum	SM 120	R	I	R	I	R	R	R	MDR	PDR	0.352	strong
Rome	CF	sputum	SM 122	S	R	S	S	R	R	R	MDR	XDR	1.300	powerful
Rome	CF	sputum	SM 123	S	I	S	S	R	R	R	MDR	XDR	0.608	strong
Rome	CF	sputum	SM 124	S	R	S	S	S	R	R	MDR		0.000	non producer
Rome	CF	sputum	SM 134	S	R	S	I	R	R	R	MDR	XDR	0.547	strong
Rome	CF	sputum	SM 135	S	R	S	I	R	R	R	MDR	XDR	0.583	strong
Rome	CF	sputum	SM 136	R	R	R	R	R	R	R	MDR	PDR	0.541	strong
Rome	CF	sputum	SM 137	S	R	S	S	R	R	R	MDR	XDR	1.123	powerful
Rome	CF	sputum	SM 138	S	R	R	S	R	I	R	MDR	XDR	0.391	strong
Rome	CF	sputum	SM 139	S	R	R	S	I	S	R	MDR		1.269	powerful
Rome	CF	sputum	SM 140	S	S	R	S	R	R	R	MDR	XDR	0.233	moderate
Rome	CF	sputum	SM 142	S	I	I	S	R	R	R	MDR	XDR	1.545	powerful
Rome	CF	sputum	SM 156	S	I	S	S	I	R	R	MDR	XDR	0.276	moderate
Rome	CF	sputum	SM 191	S	R	R	S	I	R	R	MDR	XDR	0.337	strong
Rome	CF	sputum	SM 192	R	I	R	R	R	R	R	MDR	PDR	0.446	strong
Rome	CF	sputum	SM 190	R	R	R	R	R	R	R	MDR	PDR	0.263	moderate
Rome	CF	sputum	SM 193	R	I	S	S	R	I	R	MDR	XDR	0.239	moderate
Rome	CF	sputum	SM 144	S	I	R	S	I	R	R	MDR	XDR	0.590	strong
Rome	CF	sputum	SM 130	S	R	S	S	R	R	R	MDR	XDR	0.546	strong
Rome	CF	sputum	SM 194	S	R	S	I	I	R	R	MDR	XDR	0.184	moderate
Rome	CF	sputum	SM 195	S	I	I	R	R	R	R	MDR	XDR	0.195	moderate
Rome	CF	sputum	SM 159	I	I	S	S	S	R	R	MDR		0.370	strong
Rome	CF	sputum	SM 150	S	I	S	S	R	R	R	MDR		0.391	strong
Rome	CF	sputum	SM 157	S	S	S	S	S	S	S			0.000	non producer

Abbreviations: Pescara, Santo Spirito Hospital of Pescara (Italy); Rome, Bambino Gesù Hospital, Rome (Italy); CF, cystic fibrosis; SXT, trimethoprim/sulfamethoxazole; RIF, rifampicin; CHL, chloramphenicol; LVX, levofloxacin; CIP, ciprofloxacin; TZP, piperacillin/tazobactam; MEP, meropenem; S, susceptible—standard dose; I, susceptible—increased dose (EUCAST) or intermediate (CLSI); R, resistant; MDR, multidrug resistance; XDR, extensive drug resistance; PDR, pandrug resistance.
